# Refractory pneumonia caused by *Prevotella* *heparinolytica*: a case report

**DOI:** 10.1186/s13256-024-04538-8

**Published:** 2024-04-30

**Authors:** Jiongzhou Sun, Xun Xu, Shiyuan Gao, Qiong Pan, Zian Liu, Yiwen Huang, Yixin Lian

**Affiliations:** Department of Respiratory and Critical Care Medicine, The Second Affiliated Hospital of Soochow University, Suzhou, 215004 China

**Keywords:** *Prevotellaheparinolytica*, Pneumonia, Metagenomics next-generation sequencing, Oral infections

## Abstract

**Background:**

*Prevotella* *heparinolytica* is a Gram-negative bacterium that is commonly found in the oral, intestinal, and urinary tracts. It has been extensively studied in lower respiratory tract infections in horses, which has heparinolytic activity and can secrete heparinase and further induces virulence factors in cells and causes disease. However, no such cases have been reported in humans.

**Case presentation:**

A 58-year-old male patient from China presented to the respiratory clinic in Suzhou with a productive cough producing white sputum for 20 days and fever for 3 days. Prior to this visit, a chest computed tomography scan was conducted, which revealed multiple patchy nodular opacities in both lungs. On admission, the patient presented with a temperature of 38.1 °C and a pulse rate of 110 beats per minute. Despite routine anti-infective treatment with moxifloxacin, his temperature fluctuated and the treatment was ineffective. The patient was diagnosed with *Prevotella* *heparinolytica* infection through metagenomic next-generation sequencing. Therefore, the antibiotics were switched to piperacillin–tazobactam in combination with ornidazole, which alleviated his symptoms; 1 week after discharge, the patient returned to the clinic for a follow-up chest computed tomography, and the opacities on the lungs continued to be absorbed.

**Conclusion:**

*Prevotella* *heparinolytica* is an opportunistic pathogen. However, it has not been reported in human pneumonia. In refractory pneumonia, measures such as metagenomic next-generation sequencing can be used to identify pathogens and help guide antibiotic selection and early support.

## Background

*Prevotella* *heparinolytica,* also known as *Bacteroides* *heparinolyticus*, is a Gram-negative bacterium that is exclusively anaerobic. It is commonly found in the oral, intestinal, and urinary tracts. *Prevotella* *heparinolytica* produces heparinase and hyaluronidase, which increase epithelial permeability by degrading acetylheparin sulfate in the intercellular space and bound to the epithelial basement membrane [1, 2]. The release of microbial virulence factors can contribute to the development of diseases, such as periodontal disease. Therefore, it is important to consider this factor in disease prevention and treatment.

Studies have reported that *Prevotella* *heparinolytica* is the dominant bacterium in equine lower respiratory tract infections [3]. It is believed that there is an association between this condition and oral diseases, such as periodontitis, in humans [4, 5]. However, there are no case reports of lower respiratory tract infections associated with *Prevotella* *heparinolytica* in humans. This report presents a case of pneumonia in a patient who sought medical attention due to cough and fever caused by *Prevotella* *heparinolytica*.

## Case presentation

A 58-year-old Chinese male patient presented to the respiratory clinic in Suzhou, China, with a productive cough with white sputum for 20 days and fever for 3 days. A computed tomography (CT) scan of the chest was conducted 2 weeks prior, which showed several patchy nodular opacities in both lungs. He was prescribed oral medications, including methylprednisolone 4 mg once a day and celecoxib 0.2 g once a day for 3 days. This led to some relief of his cough. Prior to admission, the patient experienced a 3-day episode of coughing with white sputum and a fever (axillary temperature of 38 °C). The patient had a history of hypertension and was taking amlodipine 5 mg once a day. He worked as a farmer and reported no history of smoking, alcohol consumption, or contact with poultry. There was no family history of genetic or psychosocial disorders or surgeries. The patient did not report any travel to epidemic areas or contact with febrile patients.

On admission on 19 February, the patient walked into the ward and was conscious. His vital signs were as follows: axillary temperature was 38.1 °C, pulse rate was 110 beats per minute, respiratory rate was 22 breaths per minute, blood pressure was 135/76 mmHg, and pulse oximetry on air was 96%. Few moist rales were heard upon auscultation of his lungs. Abdominal, neurological, and skin examinations were normal, and no heart murmur was detected during cardiac auscultation.

**Fig. 1:**
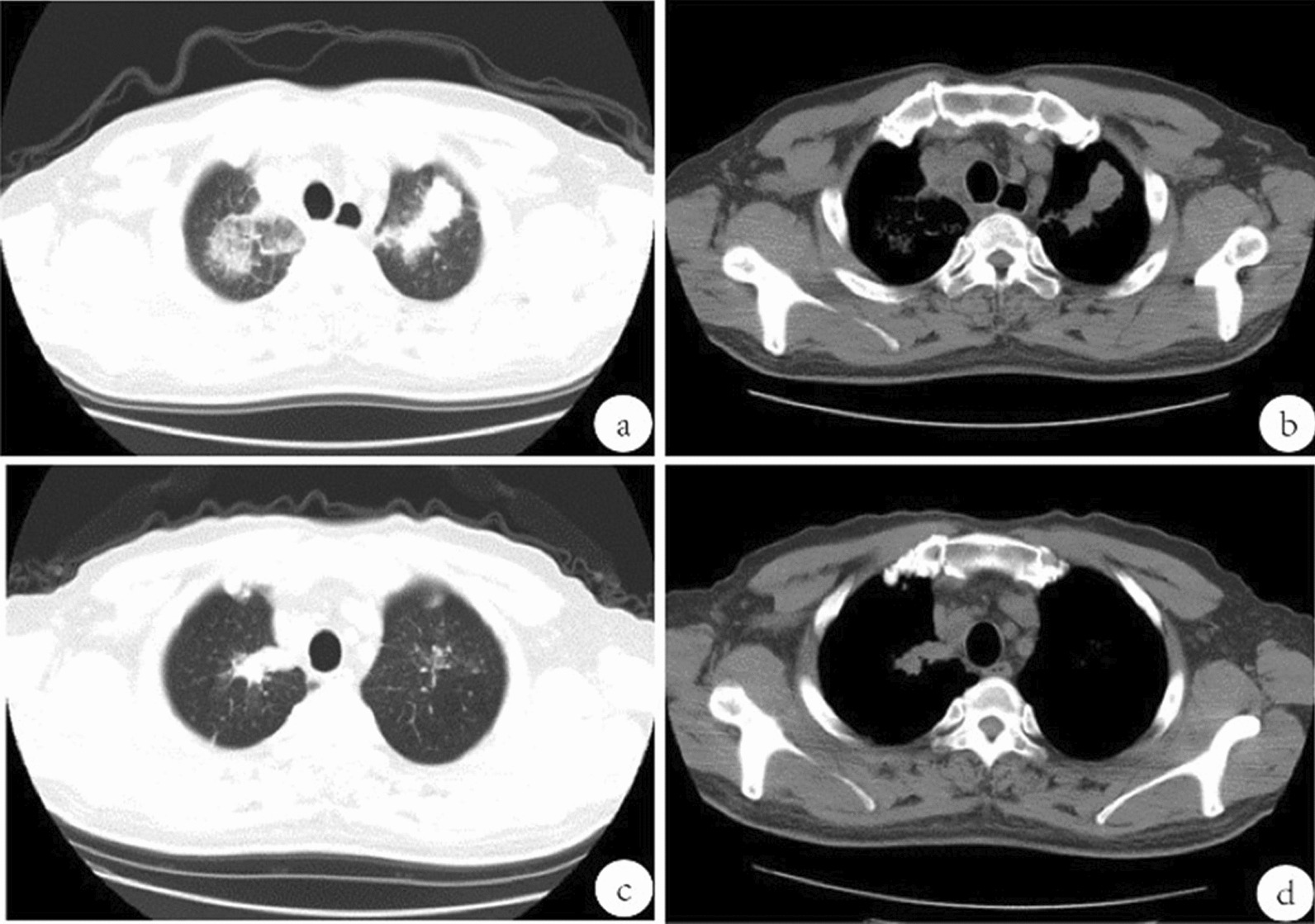
Chest computed tomography performed at different times. **a, b** Computed tomography image of the patient after admission to the hospital on hospitalization day 1 due to fever. **c, d** Computed tomography image 12 days after discharge

On the day of the presentation, a repeat chest CT revealed multiple opacities in both lungs and progression compared with previous images (Fig. 1a, b). Simultaneously, several laboratory investigations were conducted on blood and sputum samples. The patient’s white blood cell count was 21.5 × 10^9^/L (reference 3.5–9.5 × 10^9^/L), of which 88.6% were neutrophils (in absolute number 19.0 × 10^9^/L). Procalcitonin (PCT) levels were elevated, as well as d-dimer levels. The patient’s C-reactive protein (CRP) level was significantly increased at 186.9 mg/L (reference 0–10 mg/L). Bilateral blood cultures for anaerobes and aerobes were taken upon admission and yielded negative results. The results of other aetiological detection, such as the acid-fast bacillus test of sputum smear, coronavirus nucleic acid, 1,3-beta-d-glucan, galactomannan test, and antibodies for common respiratory pathogens, including *Mycoplasma* *pneumoniae*, *Chlamydia* *pneumoniae*, respiratory syncytial virus, adenovirus and coxsackievirus group B, were all negative (Table 1). The patient’s urinalysis, liver function, and renal function tests all returned normal results. Additionally, the sputum culture was negative. According to the patient’s laboratory results, which included tumour indicators, antineutrophil cytoplasmic antibodies (ANCA), and human immunodeficiency virus antibodies, there was no indication of tumours or immune system disorders at that time.

**Table 1:** Pathogens that were ruled out and test methods

Pathogen	Sample
*Bacillus* *antacidus*	Sputum
Coronavirus	Throat swab
Fungi	Blood
Aspergillus	Blood
*Mycoplasma* *pneumoniae*	Blood
*Chlamydia* *pneumoniae*	Blood
Respiratory syncytial virus	Blood
Adenovirus and Coxsackievirus group B	Blood

The patient was given intravenous moxifloxacin 0.4 g once a day as an empirical anti-infective treatment in the hospital. However, he still had a low fever with paroxysmal cough. As moxifloxacin did not alleviate the symptoms and C-reactive protein (CRP) levels remained high, a bronchoscopy was performed on 21 February. During the procedure, a brush examination was conducted on the apical and posterior segments of the right upper lobe of the lung. The samples underwent routine pathogenetic testing and metagenomic next-generation sequencing (mNGS). Results showed *Prevotella* *heparinolytica* was detected (sequence number 1620, relative abundance 55.69%) in mNGS (Table 2). The samples obtained through sterile bronchoscopy underwent a bacterial smear test, which detected Gram-negative bacilli. This result was consistent with the mNGS findings. Additionally, bronchoscopy did not reveal any evidence of neoplasm. Moreover, bacterial and fungal culture tests were conducted, all of which yielded negative results. Therefore, susceptibility testing was not performed. On the basis of the patient’s medical history, it was found that he had developed periodontitis 1 month prior. However, self-treatment with metronidazole was found to be ineffective. During the examination, it was observed that the patient had a loose left maxillary second molar. The gingiva appeared red and swollen. The patient reported pain while chewing, and the tooth was sensitive to hot and cold food. Following a dental consultation, the patient was diagnosed with periodontitis. It was noted that *Prevotella* *heparinolytica* can colonize the oral cavity. Considering the patient’s history of recurrent episodes and ineffective antibiotic therapy, it cannot be ruled out that there is a correlation between oral and pulmonary infections. On the basis of the history and examination results, it was considered that the patient has a pulmonary infection caused by *Prevotella* *heparinolytica*.

**Table 2:** Results of the patient’s metagenomic next-generation sequencing (mNGS) of bronchoalveolar lavage fluid (BALF)

Name	Sequence number	Relative abundance (%)
*Prevotella* *heparinolytica*	1620	55.69
Herpes simplex virus type I	3	0.1

**Fig. 2:**
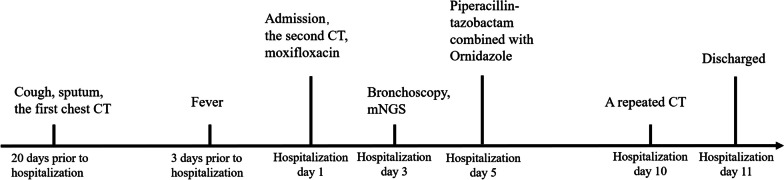
Timeline of the clinical events of the patient

Due to its higher in vivo anti-anaerobic activity, faster onset of action, and lower toxicity, the patient’s antibiotics were changed to 0.5 g of ornidazole every 12 hours combined with piperacillin-tazobactam (4.5 g per 6 hours intravenously) on 23 February for 5 days. The patient’s fever and cough symptoms gradually subsided, and his inflammatory markers, including white blood cell count (WBC, 7.3 × 10^9^/L) and CRP (15.5 mg/L), improved compared with admission. On 28 February, a chest CT revealed a marked improvement in lung inflammation. The patient was discharged on 1 March and continued oral amoxicillin 0.5 g three times daily and moxifloxacin 0.4 g once daily for 2 weeks. On 13 March, 12 days after discharge, the patient returned to the hospital for a follow-up CT (Fig. 1c, d). The inflammation in the postapical segment of the upper lobe of the right lung and the lingual segment of the upper lobe of the left lung continued to subside. Figure 2 presents the patient’s symptoms, examinations, and treatments.

## Discussion

This report describes a case of refractory pneumonia caused by *Prevotella* *heparinolytica* in a middle-aged male patient with a long-standing illness. The main symptoms were cough and fever, and imaging revealed multiple plaque-like nodules in both lungs. It is important to note that respiratory physicians may be less familiar with *Prevotella* *heparinolytica*.

*Prevotella* *heparinolytica*, once known as *Bacteroides* *heparinolyticus*, was first described in the human oral cavity by Nakamura. It is a Gram-negative anaerobe that is commonly found in the oral cavity and other tracts. It has heparinolytic activity [1, 4] and secretes a heparinase enzyme that is believed to bind and degrade acetylated heparan sulfate in the intercellular space and epithelial basement membrane. This process allows virulence factors produced by the microorganism to enter the epithelium [6].

There have been studies of horse infections with *Prevotella* *heparinolytica*, but no such cases have been reported in humans. In horse lower respiratory tract infections, specific anaerobes are important pathogens for pneumonia or pleuropneumonia, and *Prevotella* *heparinolytica* is the common dominant organism in horse pulmonary infections. A study by Yuta Kinoshita *et al*. proposed that most *Prevotella* isolates are susceptible to β-lactams and that all Bacteroides and *Prevotella* isolates are susceptible to metronidazole [3]. Laura *et al*. demonstrated that *Prevotella* spp. can produce β-lactamase and are sensitive to meropenem, piperacillin-tazobactam, chloramphenicol, and metronidazole [7]. As for *Prevotella* *heparinolytica*, it is often sensitive to metronidazole, imipenem and clindamycin. The patient’s anti-infection measures were changed to piperacillin-tazobactam combined with venous ornidazole due to its higher in vivo anti-anaerobic activity, faster onset of action, and lower toxicity.

On admission, the patient was diagnosed with community-acquired pneumonia, sepsis, and hypertension. The patient’s oral health was found to be poor despite the absence of an immune system disorder. The mNGS identified the pathogen responsible for the infection, with *Prevotella* *heparinolytica* detected. Treatment consisted of a combination of penicillin and anti-nitroimidazoles, resulting in significant improvement of the patient’s symptoms and imaging manifestations, we considered him to be infected by *Prevotella* *heparinolytica*.

For patients with refractory or severe pneumonia, early identification of pathogens is crucial for adjusting anti-infective drugs. However, the use of traditional methods, such as bacterial culture and immunological tests, is somewhat limited due to poor timeliness and low positivity rates [8]. The identification of *Prevotella* *heparinolytica* can be challenging using culture-based methods, but molecular diagnostic techniques can provide valuable insights into the detection of this pathogen [2]. Can Chang *et al*. analysed a clinical sample of 180 patients and found that the positive rate of microorganisms detected by mNGS was significantly higher compared with conventional microbiological tests [9]. Bronchoalveolar lavage fluid (BALF) is currently the recommended source of samples for testing. This helps to minimize the effects of oral colonization of bacteria and provides a representative sample of the alveolar components [10]. However, it should be noted that there are still some limitations to consider. The results may be influenced by specimen contamination, bacterial colonization, and the immunocompromised status of the patient. Therefore, in clinical applications, mNGS is significant for diagnosing diseases and guiding treatment. However, the interpretation of its results should take into account microorganisms, host factors, and the patient’s clinical manifestations.

Moreover, in the case of pulmonary infections, clinicians need to be concerned about specific pathogens, such as oral infections and oral-colonizing bacteria. It is well documented that diseases such as periodontitis and dental caries have an impact on lower respiratory tract diseases. Aspiration pneumonia is a frequent lung infection caused by oropharyngeal colonizing bacteria that enter the lower respiratory tract through inhalation, leading to disease. This is often observed in patients who are at a higher risk of aspirating oral contents, such as those with impaired consciousness [11, 12]. Patients who suffer from oral infections exhibit increased levels of hydrolases in their saliva. These enzymes can compromise the protective barrier that prevents bacterial penetration [13]. Bacteria associated with periodontal disease, such as Prevotella spp. and Clostridium spp., are thought to be involved in the progression of aspiration pneumonia [14]. Research indicates that periodontal diseases, including chronic periodontal inflammation, are prevalent in 20–50% of the global population [15]. Furthermore, chronic inflammation can spread systemically through the vascular system, potentially impacting conditions such as community-acquired pneumonia, chronic obstructive pulmonary disease, atherosclerosis, and diabetes [16]. According to some theories, systemic disease may be caused by microorganisms in local oral cavity infections due to the widespread presence of bacteria and their metabolites [17]. Numerous studies have been conducted on the correlation between the oral environment and lung infections. Patients with moderate-to-severe chronic periodontitis are at a significantly higher risk of developing community-acquired pneumonia than the general population [18]. In a follow-up study of elderly patients, researchers found an increased mortality rate from pneumonia in patients with an increased number and size of periodontal pockets compared with those without pockets [19]. Oral health has an impact on the incidence and progression of lung infections. Patients with lung infections are sometimes treated empirically due to a lack of characteristic symptoms and imaging. However, those with persistent symptoms need to be actively identified for pathogens, and early pathogenic investigations are necessary. Several studies have shown that oral bacteria can have an impact on the onset and course of lung infections [20]. Therefore, oral care plays a vital role in preventing lung infections, not only in elderly individuals, but also in the clinical management process.

## Conclusion

*Prevotella* *heparinolytica* is a low-virulence pathogen that can colonise natural cavities, such as the oral cavity, and cause lung infections. Infections typically follow a subacute course. Early diagnosis and effective anti-infection measures can lead to patient recovery. mNGS has shown high sensitivity and specificity in identifying pathogens, making it a promising diagnostic method to complement conventional microbiological tests.

## Data Availability

All data pertaining to this patient are included in this report.

## Abbreviations

CT: Computed tomography
mNGS: Metagenomics next-generation sequencing
BALF: Bronchoalveolar lavage fluid
